# A combination of l-arabinose and chromium lowers circulating glucose and insulin levels after an acute oral sucrose challenge

**DOI:** 10.1186/1475-2891-10-42

**Published:** 2011-05-06

**Authors:** Gilbert R Kaats, Samuel C Keith, Patti L Keith, Robert B Leckie, Nicholas V Perricone, Harry G Preuss

**Affiliations:** 1 Integrative Health Technologies, Inc. San Antonio, TX 78209, USA; 2 Business and Healthcare Consultants, San Antonio, TX, 78209, USA; 3 Michigan State University College of Human Medicine, East Lansing, MI, 48824-136, USA; 4 Georgetown University Medical Center, Departments of Biochemistry, Medicine and Pathology, Washington, DC, 20057, USA

## Abstract

**Background:**

A growing body of research suggests that elevated circulating levels of glucose and insulin accelerate risk factors for a wide range of disorders. Low-risk interventions that could suppress glucose without raising insulin levels could offer significant long-term health benefits.

**Methods:**

To address this issue, we conducted two sequential studies, the first with two phases. In the first phase of Study 1, baseline fasting blood glucose was measured in 20 subjects who consumed 70 grams of sucrose in water and subsequently completed capillary glucose measurements at 30, 45, 60 and 90 minutes (Control). On day-2 the same procedure was followed, but with subjects simultaneously consuming a novel formula containing l-arabinose and a trivalent patented food source of chromium (LA-Cr) (Treatment). The presence or absence of the LA-Cr was blinded to the subjects and testing technician. Comparisons of changes from baseline were made between Control and Treatment periods. In the second phase of Study 1, 10 subjects selected from the original 20 competed baseline measures of body composition (DXA), a 43-blood chemistry panel and a Quality of Life Inventory. These subjects subsequently took LA-Cr daily for 4 weeks completing daily tracking forms and repeating the baseline capillary tests at the end of each of the four weeks. In Study 2, the same procedures used in the first phase were repeated for 50 subjects, but with added circulating insulin measurements at 30 and 60 minutes from baseline.

**Results:**

In both studies, as compared to Control, the Treatment group had significantly lower glucose responses for all four testing times (AUC = *P *< 0.0001). Additionally, the Treatment was significantly more effective in lowering circulating insulin after 60 minutes from baseline (AUC = *P *= < 0.01). No adverse effects were found after acute sucrose challenge or in those who consumed LA-Cr daily for four weeks.

**Conclusions:**

As compared to a placebo control, consumption of a LA-Cr formula after a 70-gram sucrose challenge was effective in safely lowering both circulating glucose and insulin levels.

**Trial Registration:**

Clinical Trials.gov, NCT0110743

## Background

A growing body of research suggests that elevated circulating levels of glucose and insulin accelerate risk factors for a wide range of pathological disorders [1-4]. Accordingly, interventions with low-risk dietary supplements that suppress glucose levels without raising insulin levels could offer significant long-term health benefits [5]. Animal studies and a single clinical trial previously reported that consumption of l-arabinose (LA), a poorly-absorbed, readily-available sweet-tasting pentose sugar, led to significant suppression of the circulating glucose and insulin after sucrose challenge [6-8]. This appears to be related to l-arabinose's ability to lessen the rapid absorption of sucrose typically found inmodern diets thereby preventing elevation of circulating levels of glucose and insulin [5] typically found in modern diets. Similarly, other animal and human studies have also reported suppression of circulating glucose levels without elevating insulin with the consumption of various forms of chromium (Cr) [9-13]. This appears to be related to chromium's ability to enhance insulin sensitivity. The purpose of this study was to examine the effects of a formula containing l- arabinose and trivalent chromium (LA-Cr) on circulating glucose and insulin responses to sucrose challenge.

## Methods

All subjects gave written informed consent in compliance with the Helsinki Declaration as approved by the researchers' ethics committee.

### Study 1, Phase 1

A total of 20 non-diabetic subjects were enrolled in this phase from a pool of subjects who had participated in previous studies and had demonstrated a high compliance with study protocols. Their relevant characteristics are set out in Table 1. All 20 completed a DXA total body composition scan, the 50-item Quality of Life (QOL) inventory shown in Table 2 and the 43-chemistry blood test panel shown in Table 3. Blood chemistries were drawn at a Quest service Center of the subject's choice http://www.quest.com.

**Table 1:** Baseline demographics for Study 1, Phases 1 and 2, and Study 2

	Study-1, Phase-1		Study-1, Phase-2		Study 2	
Number Subjects	20		10		50	

Males	6		3		20	

Females	14		7		30	

Blacks	2		1		11	

Hispanics	3		2		12	

Whites	4		7		25	

	0		0		2	

	Mean	SD	Mean	SD	Mean	SD

Age	53.8	11.3	53.7	11.8	40.2	14.8

Weight	195.8	46.8	195.8	48.1	182.2	44.8

Height	67.3	4.1	67.3	4.2	65.8	4.4

BMI	29.9	6.0	30.2	6.1	29.6	6.0

Bone Mineral Density	1.189	0.102	1.189	0.104	1.202	0.092

% Body Fat	42.4%	7.8%	42.4%	8.1%	38.9%	9.4%

Fat Mass	83.0	22.3	83.0	22.9	70.9	28.8

Fat-free Mass	112.8	35.6	112.8	36.6	111.3	27.4

**Table 2:** Quality of Life Inventory

1	Headaches	26	Irregular heartbeat
2	Irritable bowel syndrome	27	Shortness of breath
3	Arthritis	28	Constipation
4	Premenstrual syndrome	29	Stomach gas or indigestion
5	Recurring sinus infections	30	Feeling weak
6	Tension fatigue syndrome	31	Eating too rapidly
7	Recurrent anxiety	32	Eating after being full
8	Recurrent depression	33	Embarrassed about overeating
9	Insomnia	34	Depressed over eating habits
10	Low self esteem	35	Depressed about my weight
11	Binge eating	36	Difficult to stop eating
12	Chronic tension	37	Worrying about the future
13	Lack of energy	38	Unable to concentrate
14	Food allergies	39	Forgetfulness
15	Feeling under stress	40	Bad temper or quick to anger
16	Cancer	41	Indigestion
17	Prostate problems	42	Diabetes
18	Overeating	43	Vomiting
19	Stomach pain	44	Heartburn
20	Back pain	45	Esophageal reflux
21	Pain in arms, legs, or joints	46	Control over my appetite
22	Menstrual pain or problems	47	Ability to relax
23	Chest pain	48	Heart disease
24	Dizziness	49	Fibromyalgia
25	Diarrhea	50	Difficulty in falling asleep

Subjects Rated Magnitude of Problems Occurring Over the last

30 Days Using a Scale of 0 = None, 1 = Minor, 2 = Major and 3 = Severe

**Table 3:** The 43-Panel Blood Chemistry Test Completed by Subjects in Pilot Study, Phase-1

LIPID PANEL	CBC (INCLUDES DIFF/PLT)
TRIGLYCERIDES	WHITE BLOOD CELL COUNT

CHOLESTEROL, TOTAL	RED BLOOD CELL COUNT

HDL CHOLESTEROL	HEMOBLOBIN

LDL CHOLESTEROL	HEMATOCRIT

CHOL/HDLC RATIO	MCV

METABOLIC PANEL	MCH

GLUCOSE	MCHC

UREA NITROGEN (BUN)	RDW

CREATININE	PLATELET COUNT

BUN/CREATININE RATIO	ABSOLUTE NEUTROPHILS

SODIUM	ABSOLUTE LYMPHOCYTES

POTASSIUM	ABSOLUTE MONOCYTES

CHLORIDE	ABSOLUTE EOSINOPHILS

CARBO DIOXIDE	ABSOLUTE BASOPHILS

CALCIUM	NEUTROPHILS

PROTEIN, TOTAL	LYMPHOCYTES

ALBUMIN	MONOCYTES

GLOBULIN	EOSINOPHILS

ALBUMIN/GLOBULIN RATIO	BASOPHILS

BILIRUBIN, TOTAL	OTHER MEASURES

ALKALINE PHOSPHATASE	CARDIO CRP

AST & ALT	TSH W/REFLEX TO FT4

On test day-1 (Control), after fasting for 10 hours, subjects completed a baseline "finger-stick" capillary blood sample, and consumed 70 grams of sugar dissolved in 150 grams of bottled water. Blood glucose levels were retested at 30, 45, 60 and 90 minutes. On test day 2 (Treatment), subjects followed the same procedure, but with a LA-Cr supplement containing 1,000 mg of l-arabinose and 200 mcg of a patented food-source chromium. All glucose measurements were obtained on-site using a glucometer (*ACCU-CHEK Aviva *meter, *ACCU-CHEK Multiclix*, and *Multiclix Pen*, Roche Diagnostics, Indianapolis IN).

For each subject and each timed test period, a change from baseline was obtained by subtracting the values for the corresponding baseline from the values of the four test periods. A glucose "suppression score" was obtained for each subject by subtracting the treatment change score from the control change score. The suppression scores were averaged over the 20 subjects and the changes from control scores to treatment scores were expressed as a percentage of control scores. Decreases were shown as negative percentages. The area under the curve (AUC) scores were obtained by using *KaleidaGraph*, *graphing and data analysis*, Version 3.6. The AUC scores for glucose treatment periods for each subject were compared to the control AUC scores for each subject using a paired, 2-tailed t-test. Significance was defined as *P *< 0.05.

### Study 1, Phase 2

Ten of the 20 subjects were randomly chosen and asked to consume a daily serving of LA-Cr for four weeks. Subjects provided daily tracking information on adverse effects. At the conclusion of each week, subjects completed the same Treatment sucrose challenge as described in Phase 1 and repeated the Blood, DXA, and QOL inventory at the end of the 4^th ^week.

### Study 2

Fifty new non-diabetic subjects were recruited, and completed the same DXA and QOL inventory used in phase 2 of Study 1. In addition to glucose measurements, fasting insulin measurements were also obtained at baseline, 30 and 60 minutes from baseline with and without simultaneous consumption of the LA-Cr supplement at baseline. Insulin measurements were performed by Quest Laboratories, San Antonio, TX. The same procedures and instruments used in the pilot study were used to obtain glucose and insulin suppression scores. Significance was defined as *P *< 0.05. All 50 subjects completed the glucose measurements. The phlebotomist was unable to draw blood from one subject and accordingly 49 subjects completed the insulin tests. To examine the relationship between the total glucose suppression or the total insulin suppression and baseline factors, each suppression score was compared with each baseline factor as follows: The data were ranked in order of suppression score and separated into quartiles, with Q1 representing the most suppression and Q4 representing the least suppression. An analysis of variance (ANOVA) was conducted across the 4 quartiles. Significance was defined as *P *< 0.05.

## Results

The data for each of the groups and each of the time periods are shown in Table 4. As shown in Table 5, consuming La-Cr simultaneously with a 70 gram sucrose challenge (treatment) suppressed the glucose response in both Study 1 and 2 in all four time measurements as compared to control. These differences were statistically significant for all four time periods. Circulating insulin levels were also statistically lower at 60 minutes in the treatment group. Although not shown, weekly reductions in glucose were essentially the same in each of the four weeks as were found for these subjects in the first phase of Study 1. In addition, no significant changes were found in comparisons between baseline and ending blood chemistries and self-reported QOL scores.

**Table 4:** Capillary Glucose and Venous Insulin Levels After a 70 g Sucrose Challenge With and Without Simultaneous Consumption of LA-Cr for the Pilot and Clinical Studies

Pilot Study N = 20 (Glucose Measurements Only)					
Minutes from Baseline (glucose)	:0	:30	:45	:60	:90

Mean glucose levels in the control group (sugar only)	100.4	157.9	161.3	151.4	121.5

Standard deviations of glucose in control group (sugar only)	16.1	23.8	18.5	24.7	17.0

Mean glucose levels in the treatment group	104.5	149.1	149.5	133.4	116.4

Standard deviations of glucose levels in treatment group	12.0	15.9	16.8	20.7	13.7

					

Clinical Study N = 50 (Glucose measurements)					

Minutes from Baseline (glucose)	:0	:30	:45	:60	:90

Mean glucose levels in the control group (sugar only)	97.2	150.3	151.4	141.8	120.5

Standard deviations of glucose in control group (sugar only)	10.3	22.2	25.0	25.6	22.2

Mean glucose levels in the treatment group	99.9	142.8	140.0	133.5	116.9

Standard deviations of glucose levels in treatment group	11.5	15.8	16.8	19.1	18.8

					

Clinical Study N = 49 Insulin measurements)					

Minutes from Baseline (insulin)	:0	:30	n/a	:60	n/a

Mean insulin levels in the control group (sugar only)	4.4	32.3		32.2	

Standard deviations of insulin in control group (sugar only)	3.9	24.7		19.9	

Mean insulin levels in the treatment group	4.4	29.0		24.4	

Standard deviations of insulin scores in thetreatment group	3.9	20.6		16.5	

**Table 5:** Changes from Baseline in Capillary Glucose and Venous Insulin Levels After a 70 g Sucrose Challenge With and Without Simultaneous Consumption of LA-Cr for Pilot Study (N = 20) and Clinical (N = 50).

Pilot Study N = 20 (Glucose Only)					
**Minutes from Baseline**	**30**	**45**	**60**	**90**	**AUC**

% Difference Between Treatment vs Control	-22.3%	-26.0%	-43.2%	-43.5%	-31.4%

Significance Levels	P < 0.007	P < 0.001	P < 0.001	P < 0.031	P < 0.0001

					

**Clinical Study N = 50 (Glucose Only)**					

**Minutes from Baseline (Glucose)**	**30**	**45**	**60**	**90**	**AUC**

% Difference Between Treatment vs Control	-19.1%	-26.1%	-24.8%	-27.1%	-18.4%

Significance Levels	P < 0.01	P < 0.001	P < 0.01	P < 0.05	P < 0.0001

**Clinical Study N = 49 (Insulin Only)**					

**Minutes from Baseline (Insulin)**	**30**		**60**		**AUC**

% Difference Between Treatment vs Control	-11.9%		-28.3%		-28.3%

Significance Levels	NS		P < 0.001		P < 0.01

*P *values are from repeated measures t-test and Ar

To further examine the association between suppression scores and baseline measures, glucose and insulin suppression scores were divided into four equal quartiles. An ANOVA revealed that there were no statistically significant relationships between glucose suppression scores and baseline measures of glucose, insulin, age, gender, ethnicity, scale weight, height, bone mineral density, total body fat, total body lean, and body mass index. However, there was a significant association between glucose suppression scores and % body fat (*P *= 0.038). A further comparison of the quartiles of glucose suppression scores and % body fat revealed that the greater the suppression score, the lower the % body fat (Q4 = 42.5%, Q3 = 40.8%, Q2 = 40.0%, Q1 = 32.5%). A Student t-test between the highest (n = 12) and lowest (n = 12) glucose suppression quartiles was also significant (*P *= 0.025).

## Discussion

This study compared the acute effects of the simultaneous ingestion of a combined l-arabinose and trivalent chromium formulation (LA-Cr) after a 70 gram oral challenge of sucrose. Sucrose absorption was estimated by the appearance of increased levels of circulating glucose after the sucrose challenge [6]. Data from two separate studies found an 18% to 31% reduction in glucose when taking LA-Cr supplement compared to ingesting the sucrose alone. In the second study, we also found a 28% reduction in circulating insulin concentrations 60 minutes after taking the formulation. With regard to safety, other than some discomfort with the capillary measurements, no adverse effects were reported. Nor were any adverse effects reported among the 10 subjects who consumed the LA-Cr daily for the 4-week study period.

When the effects of the LA-Cr were measured weekly with the acute oral sucrose challenge, the glucose-lowering response of the combination remained over the 28-day period. Additionally, there were no significant differences between baseline and ending values on any of the 43 blood chemistries, DXA body composition measures, or the self-reported Quality of Life Inventory after using the LA-Cr daily for 28 days.

We devised our protocol with the thought that we were essentially examining the l-arabinose in the formula. Findings similar to ours have been reported in a well-controlled rat model, i.e., l-arabinose works quickly when taken prior to a sucrose challenge and continues to work effectively over a sub chronic period of time that may provide insights into the mechanisms of action. The data support the hypothesis that l-arabinose worked by blocking sucrose absorption [6,13]. In rats, l-arabinose did not influence circulating glucose levels when no sucrose, but rather saline, was given. Under these circumstances, it did not lower glucose via enhancing uptake or metabolism of glucose. Further, l-arabinose did not affect glucose appearance when glucose replaced sucrose as the challenging sugar. Finally, *in vitro *studies have shown that l-arabinose blocks sucrase in an uncompetitive manner [14].

This was unlike effects with chromium that influence circulating glucose levels through an ability to enhance insulin sensitivity and its removal from the circulation. While chromium could have influenced the results of our sub-chronic study, it is unlikely to have done so in the acute studies since chromium does not work acutely after initial intake [8-12]. Our studies examined the product with both ingredients without partitioning the individual or interactive effects of chromium and l-arabinose.

To explore individualized reactions, we examined the association between suppression scores and baseline measures by sub-grouping glucose and insulin suppression scores into four equal quartiles. An ANOVA revealed that there were no statistically significant relationships between glucose suppression scores and baseline measures of glucose, insulin, age, gender, ethnicity, scale weight, height, bone mineral density, total body fat, total body lean, and body mass index. However, there was a significant association between glucose suppression scores and % body fat (*P *= 0.038). A further comparison of the quartiles of glucose suppression scores and % body fat revealed that the greater the suppression score, the lower the % body fat (Q4 = 32.5%, Q3 = 40.8%, Q2 = 40.8%, Q1 = 42.5%). A Student t-test between the highest (n = 12) and lowest (n = 12) glucose suppression quartiles was also significant (*P *= 0.025). This could suggest that the higher the subject's % fat, the more LA-Cr may be required to obtain the same glucose suppression results.

An ANOVA of the insulin suppression score quartiles failed to reach statistical significance on any of the baseline measures, including % body fat. However, a t-test between the highest and lowest age quartiles (Q4 = 33.9 yrs, Q3 = 40.1 yrs, Q2 = 40.6 yrs, Q1 = 46.5 yrs) revealed a significant relationship between age and insulin suppression suggesting the insulin suppression effect may be greatest in younger people. However, the irregular pattern of Q2-Q4 calls this interpretation into question, suggesting it may be a statistical artifact as a function of the multiple ANOVA analyses conducted.

The data from these two separate studies reveal that a formula containing l-arabinose and chromium (LA-Cr) can facilitate a consistent suppression of both circulating glucose and insulin without adverse side effects. The replication of the suppressive effect observed in the two sequential studies increases the confidence of the formula's efficacy. Furthermore, the percentage of subjects for whom the supplement had at least some suppressive effect, 78% for glucose and 70% for insulin, is particularly noteworthy since we had little control over how many subjects actually fasted for the required 10 hours prior to being tested.

There is a widespread belief that we are undergoing a global "epidemic" of obesity and diabetes [14-16]. Some studies have suggested that an important contributing factor is the greater intake of rapidly absorbed or simple carbohydrates, particularly sugar [17-19]. At least one study [19] suggests that rapidly absorbed carbohydrates are more harmful than those that are more slowly absorbed, perhaps due to the difference in their effects on the glucose-insulin system. The data derived from this study suggest that it may be feasible to suppress the harmful effects of glucose and insulin associated with intake of rapid carbohydrates with a low- or no-risk dietary supplement. Since even small reductions of circulating glucose and insulin can have significant health benefits, this study suggests that longer-term and dose-related studies need to be conducted.

## Abbreviations

CHO: carbohydrates; DXA: Total Body Dual-energy X-ray Absorptiometry; LA-Cr: 1.0 grams of L-arabinose and 200 μg of a patented proprietary chromium; QOL: an 86-item Quality of Life questionnaire; SG: United States Surgeon General;

## Competing interests

The authors declare that they have no competing interests.

## Authors' contributions

GRK was principal investigator contributing to the design of the study, supervision of the conduct of all testing and drafted and edited the final manuscript. HGP contributed to the study design, data interpretation, research and writing of the relevant scientific literature, and review and publication of the manuscript. HAC and NP reviewed the study design, manuscript and medical testing. SCK provided information technology support, acquired and maintained data, and provided audited data to the principle investigator. PLK reviewed and explained the informed consent form, enrolled and scheduled all subjects, supervised or conducted all DXA and glucose testing, administered on-line orders or provided subjects with requisitions for off-site blood testing, and edited and reviewed the manuscript. RBL aided in the interpretation of the data, the statistical analyses, and the preparation, editing and review of the final manuscript. All authors read and approved the final manuscript.
